# Supplementary material for: Creating unimolecular multivalent diversity in protein conjugates via the Passerini multicomponent bioconjugation with isocyanoproteins
Source: Commun Chem. 2026 Jan 22;9:46. doi: 10.1038/s42004-025-01827-1 (PMC12847923; doi:10.1038/s42004-025-01827-1)
Supplement: Supplementary file 2 — Supplementary Information [file 42004_2025_1827_MOESM2_ESM.pdf]

Supplementary Information

Creating Unimolecular Multivalent Diversity in Protein Conjugates *via* the Passerini Multicomponent Bioconjugation with Isocyanoproteins

Ana R. Humpierre,[1,2] Yanira Méndez,[1,3] Ahyoung Kim,[1] Michael Niemeyer,[1] Andrej Frolov,[1] Mirelys Saenz,[2] Raine Garrido,[4] Leslie Reguera,[1,2] Darielys Santana-Mederos,[4] Dagmar Garcia-Rivera,[4] Bernhard Westermann,[1,*] and Daniel G. Rivera[1,2,4,*]

[1]Department of Bioorganic Chemistry, Leibniz Institute of Plant Biochemistry, Weinberg 3, 06120, Halle/Saale, Germany

[2]Laboratory of Synthetic and Biomolecular Chemistry, Faculty of Chemistry, University of Havana, Zapata & G, Havana 10400, Cuba.

[3]Yusuf Hamied Department of Chemistry, University of Cambridge, Cambridge CB2 1EW, United Kingdom

[4]Finlay Institute of Vaccines, Ave 27 Nr. 19805, Havana 10600, Cuba.

*Corresponding authors: Bernhard.Westermann@ipb-halle.de (BW); dgr@fq.uh.cu (DGR)

Abbreviations

BSA, bovine serum albumin; CPs, capsular polysaccharide; DCM, dichloromethane; DIPEA, diisopropylethylamine; DMF, dimethylformamide; ESI-MS, electrospray ionization mass spectrometry; Et_3N, triethylamine; EtOAc, ethyl acetate; FA, formic acid; HPLC, High Performance Liquid Chromatography; HRMS, high resolution mass spectrometry; MALDI-TOF, Matrix-Assisted Laser Desorption/Ionization Time-of-Flight; MeOH, methanol; MW, molecular weight; NMR, nuclear magnetic resonance; PBS, phosphate buffered saline; RT, room temperature; SE-FPLC, Size-Exclusion Fast Protein Liquid Chromatography; SE-HPLC, Size-Exclusion High Performance Liquid Chromatography; SDS-PAGE, Sodium Dodecyl Sulfate Polyacrylamide Gel Electrophoresis; THF, tetrahydrofuran; TLC, thin layer chromatography.

General

All solvents were distilled following reported procedures. All commercially available reagents employed in the synthesis (reagent grade) and characterization (analysis grade) were purchased from Merck (Sigma-Aldrich), Carbolution, abcr, Carl Roth and TCI Chemicals and were used without further purification. BSA (>96%, Lot SLCB9433), RNAse A (Lot SLCB3862) and ubiquitin (>98%, Lot SLBZ2180) were purchased from Sigma-Aldrich. Natural CPs14 and CPsC were isolated from the bacteria *S. pneumoniae* and *N. meningitidis*, respectively, at dedicated production facilities of the Finlay Institute of Vaccines, Cuba. The GM3 glycolipid was also produced and provided by the Finlay Institute of Vaccines as reported in the manuscript.

NMR. ^{1}H NMR and ^{13}C NMR spectra were recorded at 500/400 MHz and 125/100 MHz, respectively, on a Bruker/Advance DRX 500 and on a Varian Mercury 400. Chemical shifts (δ) are reported in ppm relative to the TMS (^{1}H NMR) and to the solvent signal (^{13}C NMR).

Mass Spectrometry Analysis. ESI mass spectra of small molecular-weight compounds were obtained from a Fourier transform ion cyclotron resonance (FT-ICR) mass spectrometer (Agilent Technologies 1200 series), an RF-only hexapole ion guide and an external electrospray ion source. Samples were dissolved in water, methanol, acetonitrile or mixtures of them. The data was analyzed using the software Analyst® 1.6.2.

ESI-TOF measurements

ESI-ToF mass spectra were obtained from a mass spectrometer A TripleToF 6600-1 (Sciex), equipped with ESI-DuoSpray-Ion-Source (operated in positive mode) and controlled using the software Analyst

1.7.1 TF (Sciex). High resolution ESI mass spectra of organic compounds were obtained with the parameters: ion spray voltage 5.500 V, nebulizing gas 60 p.s.i., source temperature 450 °C, drying gas 70 p.s.i., curtain gas 35 p.s.i. Data acquisition was carried out on MS^1-ToF mode, with 100 a 1500 Da scan and 50 ms accumulation time. Samples were dissolved in water, methanol, acetonitrile or mixtures of them, 10 µL were applied with direct injection with methanol/water mixture or acetonitrile with 0.1% formic acid. The data was analyzed using the software PeakView 2.2.0.

For the protein samples analysis, the MS system was coupled to a UHPLC system (Dionex UltiMate 3000, Thermo Fisher Scientific), equipped with a X Bridge Protein C4 BEH column (3.5 µm particle size, 2.1 × 100 mm ID, 10-500 K, 40 °C column temperature; Waters). Water (A) and acetonitrile (B) with 0.1% formic acid were employed as mobile phase. The chromatographic separation was carried out with a 5% B initial gradient (isocratic for 1 min) and then increase up to 70% B (0.4 $mL.min^{-1}$). Samples were dissolved in water and 3-10 µL were applied. The data was analyzed using the software PeakView 2.2.0. The protein mass deconvolution was performed with the Bio Tool Kit 2.2.0 (initial mass 20 kDa and final mass 100 kDa) in the region 1000-1500 Da *m/z* of the mass spectra corresponding to the retention time 3.6-3.8 min in the chromatogram.

MALDI TOF measurements

MALDI targets plate (MTP 384 target plate ground steel BC, Bruker Daltonics, Bremen, Germany) was washed briefly with Millipore water, HPLC grade methanol and HPLC grade isopropanol using disposable wipes KIMTECH® Science lab wipes (Kimberly-Clark GmbH, Koblenz-Rheinhafen, Germany). 1 µL of the samples was spotted onto the target plate. The samples were mixed with 1 µL of a matrix solution (25 $mg.mL^{-1}$ of 2,6-dihydroxiacetophenone (DHAP) in 50% (*v/v*) ACN/0.1% (*v/v*) TFA). Mass spectra were calibrated using Protein Calibration Standard II (Bruker Daltonics, Bremen, Germany) for the mass range between 10000 and 70000 Da. Protein calibration standard (II) was prepared in 125 µL TA solvent (mixture of ACN and 0.1% (*v/v*) TFA in a volume ratio 1:2) based on the instructions from the manufacturer. Protein calibration standard II contains the following three proteins: trypsinogen, protein A and bovine serum albumin (BSA).

The measurements of protein samples were analyzed by MALDI-TOF MS (Bruker Daltomic Ultraflex III MALDI-TOF/TOF MS, Bruker Daltonics, Bremen, Germany) using flexControl 3.4. Ionization was achieved using Smartbeam II laser optics with the laser focus set to ultra with 500 Hz frequency at laser power between 40 – 60 % in a random walk raster pattern (complete sample). The instrument was operated in the positive ionization and linear mode by accumulating 1000 laser shots in the range of

m/z 10000-140000 at a detector energy of 3155 V with a detector gain of 20.0×. Samples were analyzed with optimized voltages for ion sources (ion source 1, 25 kV, ion source 2, 23.2 kV), lens (7.5 kV) and a pulsed ion extraction of 150 ns. In the range of *m/z* 3000-20000, the instrument was operated in the positive ionization and linear mode by accumulating 1000 laser shots at a detector energy of 2959 V. A digitizer setting was used at a sampling rate of 0.13 GS/s. The laser parameters were set up as follows: Global Attenuator Offset 68 %, Attenuator Offset 1 %, Attenuator Range 20 %, Focus Offset 0%, Focus Range 100% and Focus Position 8 %. Data acquisition was manually done by FlexControl 3.4 software and data processing was performed by FlexAnalysis 3.4 software (Bruker Daltonics, Bremen, Germany).

Column Chromatography

Flash column chromatography was carried out using silica gel 60 (70–230 mesh) cartridges. Analytical thin layer chromatography (TLC) was performed using silica gel aluminum sheets and visualization of the compounds with UV light and stain developers (phosphomolybdic acid, potassium permanganate or nickel chloride).

Size-Exclusion HPLC and FPLC analysis of CPs, BSA and glycoconjugates

Natural, fragmented, and oxidized CPs were analyzed in a Merck HPLC with a TSK 5000 PW column at 1.0 $ml.min^{-1}$ of flow in NaCl 0.9%, with a refractive index detector. Data was processed using Clarity Chrom software. Samples were centrifuged and dissolved at approximately 1 $mg.mL^{-1}$ in PBS pH 7.4.

The unmodified BSA and its glycoconjugates were analyzed in an ÄKTA pure 25 FPLC with a Superose® 6 Increase 10/300 GL column at 0.5 mL/min in PBS pH 7.4 (0.01 $mol.L^{-1}$) and 5 °C, with a UV detector at 280 nm. Data was processed using the Unicorn 7.3 software. Samples were centrifuged and dissolved at approximately 1-2 $mg.mL^{-1}$ of protein concentration in PBS pH 7.4. In the case of the glyconjugates, two fractions (fraction 1: 6.7 - 9.7, fraction 2: 9.7 – 15.0 mL) were separated and subsequently analyzed employing the same conditions mentioned above.

Supplementary Methods

Colorimetric determinations

Carbohydrate and protein contents were determined using different colorimetric techniques. The sulfuric acid-orcinol method was employed for the carbohydrate content determination in CPs 14, while a periodate-resorcinol method was employed for the carbohydrate content determination of CPs MenC. A modified Park-Johnson method was employed for the quantification of carbonyl groups in periodate-

oxidized CPs14. The Bradford´s method was employed for BSA protein quantification, while the BCA method was employed for RNAse A and Ubiquitin protein quantification. Colorimetric determinations were performed using a spectrophotometer Jenway 6705 UV/Vis and a plate reader spectrophotometer SpectraMax M5.

Bradford method

Seven standard solutions containing BSA with concentrations 20, 30, 40, 50, 60, 80, 100 µg/mL were prepared from a solution of 400 µg/mL to construct a calibration curve. 50 µL of each standard solution, the blank and the samples – in triplicate – were pipetted into wells of 96-well plates. Then, 200 µL of 5-times diluted ROTI®Quant reagent (Carl Roth) were added to the wells containing the standards, blank and samples. The plate was incubated for 5 min at room temperature and the absorbance was measured at 595 nm.

Bicinchoninic acid method

Five standard solutions containing BSA with concentrations 200, 400, 600, 800, 1000 µg/mL were prepared from a solution of 2 $mg.mL^{-1}$ to construct a calibration curve. 25 µL of each standard solution, the blank and the samples – in triplicate – were pipetted into wells of 96-well plates. Then, 200 µL of a BCA solution were added to the wells containing the standards, blank and samples. The plate was incubated for 30 min at 37 °C and the absorbance was measured at 562 nm.

Sulfuric acid-orcinol method

Macroassay in test tubes

Seven standard solutions containing anhydrous D-(+)-glucose with concentrations 0.05; 0.02; 0.10; 0.20; 0.40; 0.60; 0.80 $mg.mL^{-1}$ were prepared from a stock solution of 1 $mg.mL^{-1}$ to make a calibration curve. 100 µL were taken from the different samples, the standard solutions and the blank. 200 µL of a solution containing 2% of orcinol and a 25% H_2SO_4 aqueous solution were added, and the mixtures were stirred vigorously. Then, 1.5 mL of a 60% H_2SO_4 were added and after stirring vigorously, the mixtures were heated up to 80 °C for 20 min and the absorbance was read at 530 nm.

Microassay in 96-well plates

Seven standard solutions were generated by adding into wells 4, 8, 12, 16, 20 µg of an anhydrous D-(+)-glucose solution (2 $mg.mL^{-1}$) and bringing to a final volume of 30 µL to construct a calibration curve. 30 µL of blank and the samples – in triplicate – were pipetted into wells. Then, 150 µL of concentrated sulfuric acid were added to the wells containing the standards, blank and samples; and

the plate was incubated for 15 min at 90 °C protected from light. After this time, 30 μL of an orcinol-sulfuric solution were added and the plate was incubated for 5 min at room temperature. The absorbance at 490 nm was immediately read.

Resorcinol method

Macroassay in test tubes

Six standard solutions containing *N*-acetylneuraminic acid with concentrations 5; 10; 15; 20; 40 and 60 μg.mL^{-1} were prepared from a solution of 1 mg.mL^{-1} to construct a calibration curve. 200 μL were taken from the different samples, the standard solution and the blank. 500 μL of a solution containing 2% of resorcinol were added and the mixtures were stirred vigorously. The mixtures were heated up to 100 °C for 15 min and then cooled down in an ice bath for 10 min. Then, 5 mL of 95% *tert*-butyl alcohol were added and the mixtures were stirred again. The absorbance was read at 620 nm.

Microassay in 96-well plates

Six standard solutions were generated by adding into wells 2, 4, 6, 8, 10, 12 μg of an *N*-acetylneuraminic acid solution (2 mg.mL^{-1}) and bringing to a final volume of 30 μL to construct a calibration curve. 30 μL of blank and the samples – in triplicate – were pipetted into wells. Then, 10 μL of the periodic acid solution were added to the wells containing the standards, blank and samples; and the plate was incubated in an ice box for 30 min protected from light. After, 100 μL of the resorcinol solution were added and the plate was incubated for 30 min at 90 °C. Then, the plate was cooled down and 100 μL of *tert*-butyl alcohol were added. The absorbance was read at 620 nm.

Modified Park-Johnson Method

A calibration curve was constructed preparing solutions of anhydrous D-(+)-glucose with concentrations 5, 10, 20, 30, 40, 50, 60 nmol/mL. 200 µL were took from the samples, standard, or blank and 200 μL of a solution of carbonate-cyanide (0.0015 mol.L^{-1} of potassium ferricyanide, Fluka 98% and 0.05 mol/L of sodium carbonate, Merck 99 %) were added and the mixtures were stirred vigorously. Then, 200 μL of a solution of 0.01 mol.L^{-1} of a potassium hexacyanoferrate solution (Merck 99%) were added. After stirring vigorously, the mixtures were heated up to 100 °C for 15 min, left to reach room temperature and then treated with 1mL of a solution of ammonium and iron (III) sulfate (5.6 mmol.L^{-1}, Merck 99 %). The samples were heated up to 50 °C for 15 min; left to reach room temperature and then 200 μL of an oxalic acid solution were added. After stirring vigorously, the

absorbance was read at 690 nm. The results were expressed as moles of carbonyl groups per repetitive unit (C=O/RU).

Isocyanide identification using Ni(II)-staining

A 2% solution of $NiCl_2.6H_2O$ in ethanol was used for the qualitative identification of isocyanides on TLC, which is based on the reaction of Ni(II) ions with the isocyanide group to form colored coordination complexes. The TLC plate is immersed in the Ni(II) solution and then warmed gently with a heating gun until the appearance of a reddish-brown or violet-colored spot, which indicates the presence of an isocyanide group in the molecule.

Electrophoresis on polyacrylamide gel (SDS-PAGE)

In the case of samples containing BSA, a separating gel with 10% of polyacrylamide was used, while in the case of samples containing RNAseA and Ubiquitin a separating gel with 15% of polyacrylamide was used. In all cases, a resolving gel with 5% of polyacrylamide was used. All the samples were dissolved in Laemmli buffer and heated in a heating block at 95 ^{o}C for 5 min, and then spun down. The experiments were run at 150 mV. In order to identify the proteins, 5 µg of BSA and 8 µg of RNAse A and Ubiquitin were applied, and a Coomassie staining protocol was used. To visualize the carbohydrate, 10 µg of the protein glycoconjugate were applied and a Fuchsin staining protocol was used. To visualize the fluorescent tag in the labelled proteins, the gel was visualized by in-gel fluorescence scanning using a Typhoon 9410 Imager (GE Healthcare) and stained with Coomassie afterwards.

Synthesis of isocyanide 1a

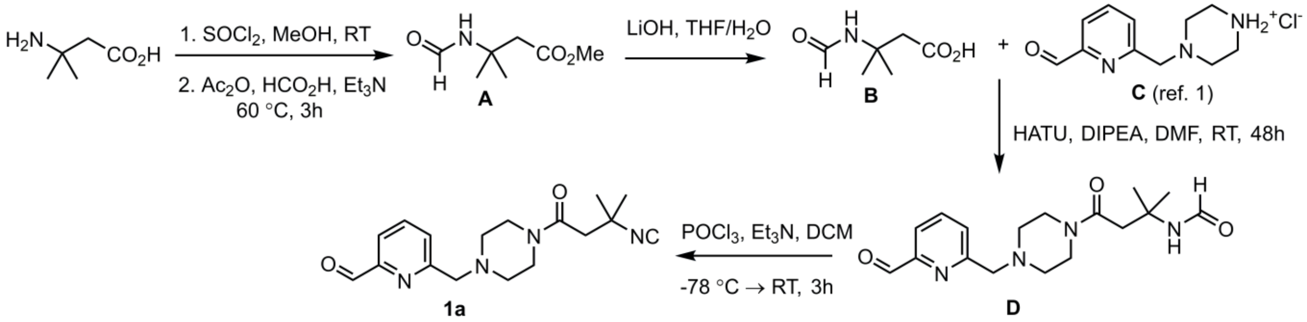

Intermediate A. 3-amino-3-methylbutanoic acid (1.17 g, 0.01 mol) was dissolved in 100 mL methanol and the solution was cooled to 0 °C. Thionyl chloride (2.2 mL, 3 equiv) was added dropwise and the reaction mixture was stirred for 12 h at room temperature. The solvent was removed under reduced pressure and the corresponding methyl ester was precipitated and washed with cold ether to obtain the

corresponding methyl ester (R_f 0.69, DCM/MeOH/FA 1.6:0.4:0.05). Acetic anhydride (9.45 mL, 0.1 mol, 10 equiv) and formic acid (5.65 mL, 0.15 mol, 15 equiv) were reacted for 3 h at 60 °C and this mixture was slowly added to a stirring solution of the methyl ester obtained before and Et_3N (22 mL, 0.16 mol) in 80 mL of THF at 0 °C. The reaction mixture was allowed to warm to room temperature, stirred for 12 h and then diluted with 250 mL of EtOAc. The organic phase was washed with water (3×50 mL) and brine (50 mL), and then dried over anhydrous Na_2SO_4 and concentrated to dryness to produce the corresponding formamide **A** (1.47 g, 93% yield, R_f 0.23 *n*-hexane/EtOAc 1:1), which was used without further purification and characterization.

Intermediate B. Crude intermediate **A** was dissolved in 50 mL of THF/H_2O 2:1 and the solution was cooled in an ice bath. LiOH (1.1 g, 3 equiv) was added and the mixture stirred at room temperature for 3 h. Then, the pH was adjusted to 3 using a 1N aqueous HCl solution and the product was extracted EtOAc (2×80 mL). The organic phase was washed with - brine (50 mL) and dried over Na_2SO_4 to obtain intermediate **B** (1.2 g, 90% yield, R_f 0.32, DCM/EtOAc 1:1), which was used without further purification and characterization.

Intermediate C. This compound was produced exactly as reported by Francis and co-workers,[1] and it was used without further purification and characterization.

Isocyanide 1a. Intermediate **B** (300 mg, 2 mmol) and **C** (492 mg, 2 mmol) were dissolved in 10 mL of dry DMF and the solution was cooled to 0 °C and treated with HATU (850 mg, 2.2 mmol) and DIPEA (0.78 mL, 4.5 mmol). The reaction mixture was allowed to warm to room temperature and stirred for 48 h. The mixture was diluted with 200 mL of EtOAc and the organic phase was washed with water (3×50 mL), brine (50 mL), dried over anh. Na_2SO_4 and concentrated to dryness. The residue was purified by flash column chromatography with EtOAc/Et_3N/MeOH 92:4:4 to afford the formamide **D** (501 mg, 76% yield, R_f 0.34, EtOAc/Et_3N/MeOH 92:4:4, used without further characterization). Formamide **D** (501 mg, 3.1 mmol) was dissolved in 50 mL of dry DCM and Et_3N (1.42 mL, 4.75 mmol) and cooled to -78 °C in a dry ice-acetone bath. $POCl_3$ (0.21 mL, 2.25 mmol) was added dropwise and the reaction mixture was stirred under nitrogen atmosphere. After 1 h, the dry ice-acetone was removed and the reaction was stirred and allowed to warm room temperature for 3 h. The residue was poured into 50 mL of cold water and extracted twice with 100 mL of DCM. The organic phase was washed with water (2×50 mL) and brine, and then dried over anhydrous Na_2SO_4 and concentrated to dryness. The crude was purified by flash column chromatography with EtOAc/Et_3N/MeOH 92:4:4 to afford isocyanide **1a** as a pale yellow syrup (203 mg, 52% yield, R_f 0.30, EtOAc/Et_3N/MeOH 92:4:4,

isocyanide identification by Ni(II) staining). ^{1}H NMR (400 MHz, $CDCl_3$): δ 1.50 (s, 6H), 2.50 (t, *J* = 8.0 Hz, 2H), 2.76 (s, 2H), 3.47 (t, *J* = 7.9 Hz, 2H), 3.78 (s, 2H), 7.70 (t, *J* = 4.3 Hz, 1H), 7.86 (d, *J* = 4.1 Hz, 2H), 10.07 (s, 1H). ^{13}C NMR (100 MHz, $CDCl_3$): δ 28.4, 47.3, 53.1, 60.0, 120.3, 123.5, 125.8, 127.3, 137.3, 137.4, 152.3, 155.0, 159.4, 193.5. ESI-HRMS: calcd. for $C_{17}H_{22}N_4O_2$: 313.1700 $[M-H]^-$; found: *m/z* 313.1670 $[M-H]^-$.

Synthesis of isocyanide 1b

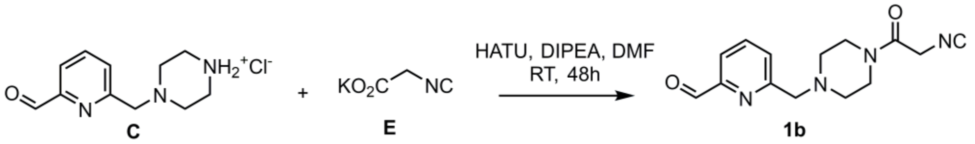

Methyl isocyanoacetate (149 mg, 1.5 mmol) was dissolved in a 10 mL of THF/H_2O 2:1 (*v/v*) and the solution was treated with KOH (83 mg, 1.5 mmol). The reaction mixture stirred at room temperature for 3 h, then the organic solvent was evaporated under reduced pressure and the remaining water was lyophilized to obtain potassium isocyanoacetate **E** as a yellow amorphous solid (185 mg). This latter compound (1.5 mmol) was dissolved in 5 mL of dry DMF and the solution was cooled to 0 °C and treated with HATU (435 mg, 1.5 mmol) and DIPEA (0.52 mL, 3 mmol). The stirring solution was treated with compound **C** (500 mg, 2 mmol) and the reaction mixture was stirred for 48 h at room temperature. The mixture was diluted with 100 mL of EtOAc and the organic phase was washed with water (3×30 mL), brine (30 mL), dried over anh. Na_2SO_4 and concentrated to dryness. The residue was purified by flash column chromatography with EtOAc/Et_3N/MeOH 96:2:2 to afford isocyanide **1b** as a pale yellow syrup. R_f 0.32, EtOAc/Et_3N/MeOH 92:4:4, isocyanide identification by Ni(II) staining. ^{1}H NMR (400 MHz, $CDCl_3$): δ 2.46 (t, *J* = 9.9 Hz, 2H), 3.44 (t, *J* = 9.9 Hz, 2H), 3.73 (s, 2H), 4.07 (s, 2H), 7.52 (b, *J* = 7.7 Hz, 1H), 7.56 (b, *J* = 7.7 Hz, 1H), 7.74 (d, *J* = 7.7 Hz, 2H), 10.07 (s, 1H). ^{13}C NMR (100 MHz, $CDCl_3$): δ 44.7, 52.9, 63.9, 123.3, 123.8, 136.9, 151.7, 154.7, 157.1, 193.3. ESI-HRMS: calcd. for $C_{15}H_{17}N_3O_2$: 271.1321; found: *m/z* 272.1399 $[M+H]^+$.

Synthesis of isocyanide 4

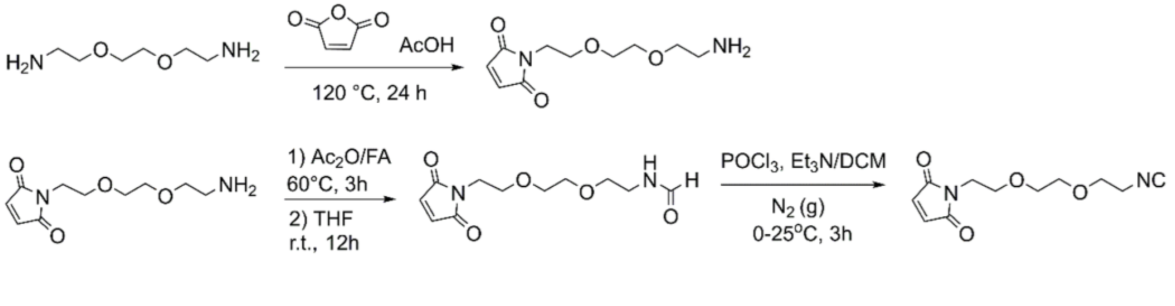

Isocyanide 4. 2,2´-(ethylenedioxy)bis(ethylamine) (2.96 g, 0.02 mol) and maleic anhydride (0.49 g, 0.005 mol) were suspended in 50 mL of acetic acid and stirred under reflux at 120 °C for 24 h. Then, the mixture was cooled down to room temperature, cooled in an ice bath and neutralized carefully with sat. aq. $NaHCO_3$. The crude was extracted with a mixture of chloroform and methanol (2×10 mL, 10:1, *v/v*), and the organic layer was washed with sat. aq. $NaHCO_3$ and brine, and then dried over anh. Na_2SO_4 and rotoevaporated to produce 2-(2-(2-meleimidoethoxy)ethoxy)ethan-1-amine (0.89 g, 78% yield, R_f 0.35, EtOAc/MeOH 3:1). This intermediate (0.89 g, 0.004 mol) was subjected to the formylation and dehydration procedure described above to produce a crude product, which was purified by flash column chromatography with *n*-hexane/EtOAc 1:1 to obtain the pure isocyanide **4** as pale yellow amorphous solid (0.63 g, 64% yield, R_f 0.32, *n*-hexane/EtOAc, 1:1). ^{1}H NMR (400 MHz, $CDCl_3$): δ 3.55 (t, J = 5.6 Hz, 2H), 3.62-3.69 (m, 8H), 3.74 (m, 2H); 6.71 (s, 2H). ^{13}C NMR (100 MHz, $CDCl_3$): δ 37.1, 41.7, 68.0, 68.7, 70.1, 70.8, 134.2, 157.2, 170.6. ESI-HRMS: calcd. for $C_{11}H_{15}N_2O_4$: 239.1032 $[M+H]^-$; found: *m/z* 239.1027 $[M+H]^-$.

Synthesis of isocyanide 11b

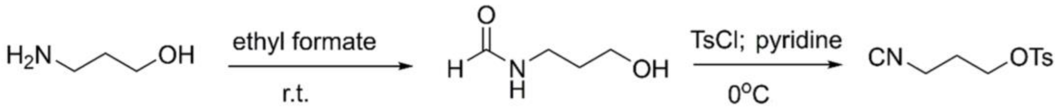

Isocyanide 11b. 3-amino-1-propanol (0.75 g, 0.01 mol) was dissolved in 50 mL of ethyl formate and the reaction mixture was stirred at room temperature for 6 h. The solvent of the reaction was removed under reduced pressure and the residue was distilled under vacuum to obtain the corresponding formamide (85% yield). The formamide was reacted with *p*-toluenesulphonyl chloride (1.1 equiv) in pyridine in an ice bath for 1 h until the reaction was completed, as indicated by TLC. The crude product was treated with cold water (50 mL) and extracted with diethylether/*n*-hexane (5:1, *v/v*); the organic layer was dried on Na_2SO_4 and rotoevaporated. The residue was purified by flash column chromatography with *n*-hexane/EtOAc (gradient 4:1 to 3:1) to obtain the 3-isocyanopropyl 4-methylbenzenesulfonate **11b** as pale brown amorphous solid (1.7 g, 71% yield). R_f 0.14 *n*-hexane/EtOAc 4:1. ^{1}H NMR (400 MHz, $CDCl_3$): δ 2.03 (m, 2H), 2.46 (s, 3H), 3.49 (t, J = 6.4 Hz, 2H), 4.17 (t, J = 5.7 Hz, 2H),7.38 (d, J =8.1 Hz, 2H), 7.80 (d, J = 8.3 Hz, 2H). ^{13}C NMR (100 MHz, $CDCl_3$): δ(ppm) = 21.7, 28.7, 37.7, 65.9, 127.9, 130.0, 132.4, 145.3, 157.6. ESI-MS: $[M+H]^+$ 240.1; $[M+NH_4]^+$ 257.2; $[M+Na]^+$ 262.0; $[M+K]^+$ 278.1. ESI-HRMS: calcd. for $C_{11}H_{13}NO_3S$: 240.29 $[M+H]^-$; found: *m/z* 240.0690 $[M+H]^-$.

Synthesis of isocyanide 11a

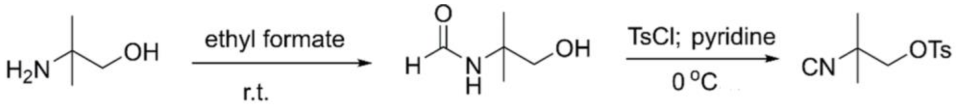

Isocyanide 11a. 2-isocyano-2-methylpropyl 4-methylbenzenesulfonate (**11a**) was synthesized from 2-amino-2-methylpropanol (0.01 mol) following the procedure described above (67% yield, as pale brown amorphous solid). R_f 0.34 *n*-hexane/EtOAc 4:1). ^{1}H NMR (400 MHz, $CDCl_3$): δ 1.43 (s, 6H), 2.47 (s, 3H), 3.90 (s, 2H), 7.38 (d, *J* = 8.1 Hz, 2H), 7.82 (d, *J* = 8.3 Hz, 2H). ^{13}C NMR (100 MHz, $CDCl_3$): δ 21.7, 25.7, 56.0, 74.0, 128.0, 130.1, 132.2, 145.4, 156.6. HR-ESI-MS: calcd. for $C_{12}H_{16}NO_3S$: 254.0851 $[M+H]^-$; found: *m/z* 254.0852 $[M+H]^-$.

Synthesis of isocyanide 13

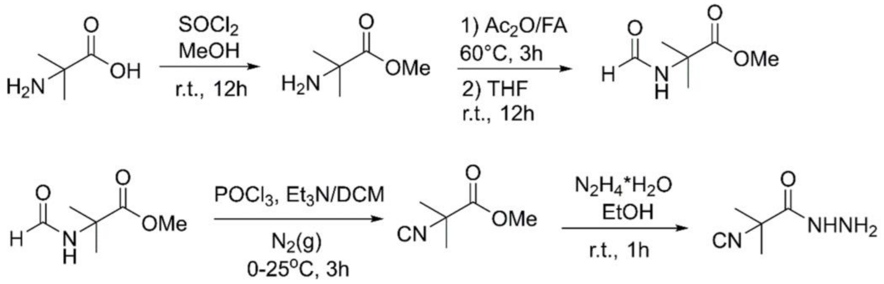

Isocyanide 13. α-Amino-isobutyric acid (1 g, 0.01 mol) was dissolved in 50 mL of methanol and thionyl cloride was added (2.2 mL, 0.03 mol, 3 equiv) to the solution at 0 °C. The reaction mixture was stirred for 12 h at room temperature. The solvent was removed under reduced presure and the product was precipitated from cold ether and washed to obtain the corresponding methyl ester (1.27 g, R_f 0.69, DCM/MeOH/FA 1.6/0.4/0.05). ***Formylation procedure.*** Acetic anhydride (9.45 mL, 0.1 mol, 10 equiv) and formic acid (5.65 mL, 0.15 mol, 15 equiv) were reacted for 3 h at 60 °C and this mixture was slowly added to a stirring solution of α-amino-isobutyric methyl ester and Et_3N (22 mL, 0.16 mol) in 80 mL of THF at 0 °C. The reaction mixture was allowed to warm to room temperature, stirred for 12 h, and then diluted with 250 mL of EtOAc. The organic phase was washed with water (3×50 mL) and brine (50 mL), and then dried over anhydrous Na_2SO_4 and concentrated to dryness to produce the corresponding formamide (R_f 0.23, *n*-hexane/EtOAc 1:1) (1.36 g, 94% yield). ***Dehydration procedure.*** The formamide (0.0094 mol) was dissolved in 30 mL of dry DCM and Et_3N (6.5 mL, 0.047 mol, 5 equiv) and cooled in an ice bath. $POCl_3$ (1.05 mL, 0.0113 mol, 1.2 equiv) was added and the reaction mixture was stirred under nitrogen atmosphere for 3 h at room temperature. The residue was poured into 50 mL of cold water and extracted with DCM (100 mL). The organic phase was washed with water

(2×50 mL) and brine (50 mL), and then dried over anhydrous Na_2SO_4 and concentrated to dryness. Flash column chromatography purification with *n*-hexane/EtOAc (10:1 to 6:1) produced the pure methyl 2-isocyano-2-methylpropanoate (0.70 g, 52% yield, R_f 0.81, *n*-hexane/EtOAc 1:1). ***Hydrazide formation.*** This latter compound was dissolved in ethanol (50 mL) and treated with hydrazine monohydrate (0.75 mL, 3 equiv). The mixture was stirred at room temperature for 4 h and concentrated to half volume. The product was precipitated by the addition of cold ether to obtain the pure 2-isocyano-2-methylpropanehydrazide (**13**) (0.42 g, 31% overall yield). ^{1}H-NMR (500 MHz, DMSO-d$_6$): δ 1.27 (s, 6H), 6.98, 6.80 (2H), 9.97 (1H). ^{13}C NMR (125 MHz, DMSO-d$_6$): δ 25.9, 51.9, 156.5, 163.6. ESI-HRMS: calcd. for $C_5H_{10}N_3O$: 128.0824 [M+H]$^-$; found: *m/z* 128.0823 [M+H]$^-$.

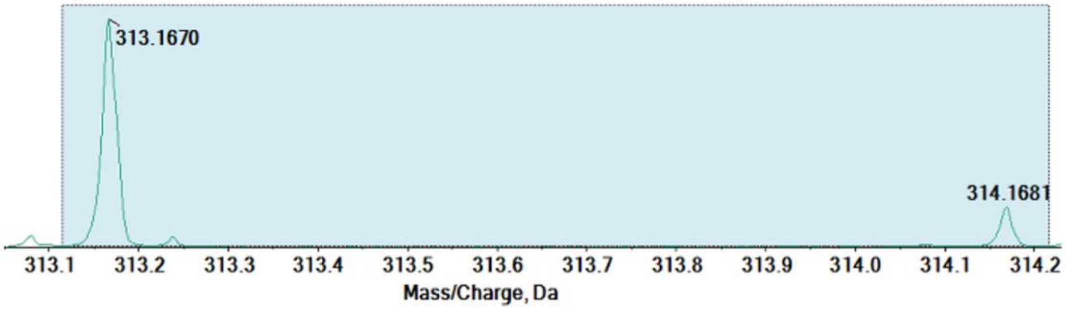

Figure S1. HR-MS spectrum of isocyanide **1a**.

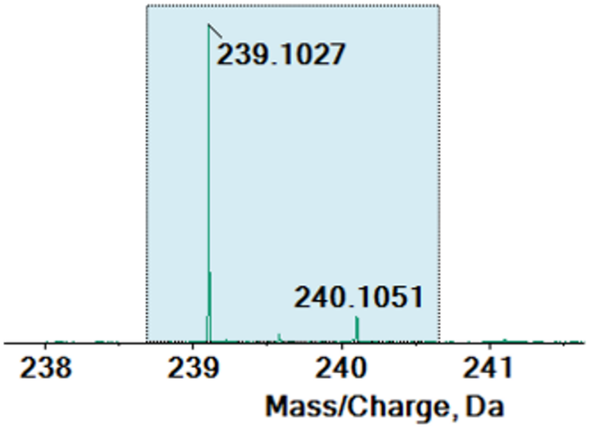

Figure S2. HR-MS spectrum of isocyanide **4**.

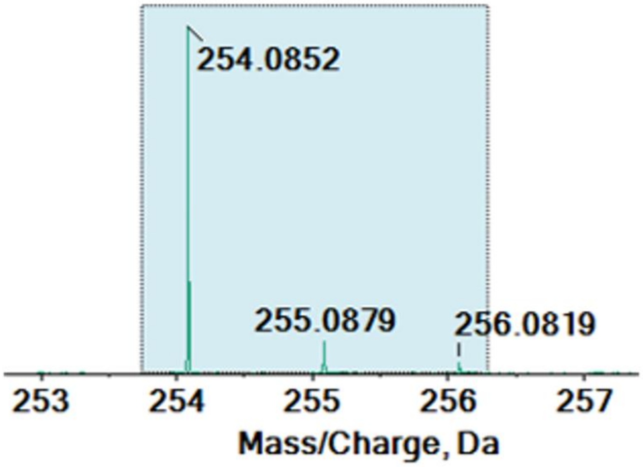

Figure S3. HR-MS spectrum of isocyanide **11a**.

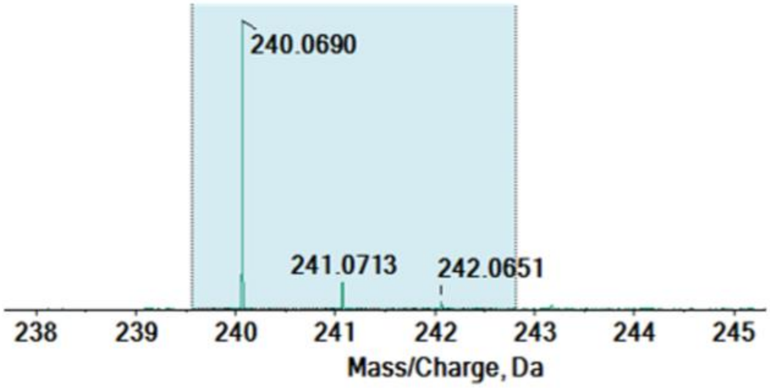

Figure S4. HRMS spectrum of isocyanide **11b**.

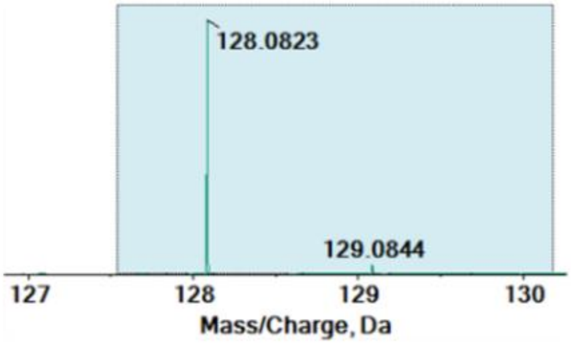

Figure S5. HR-MS spectrum of isocyanide **13**.

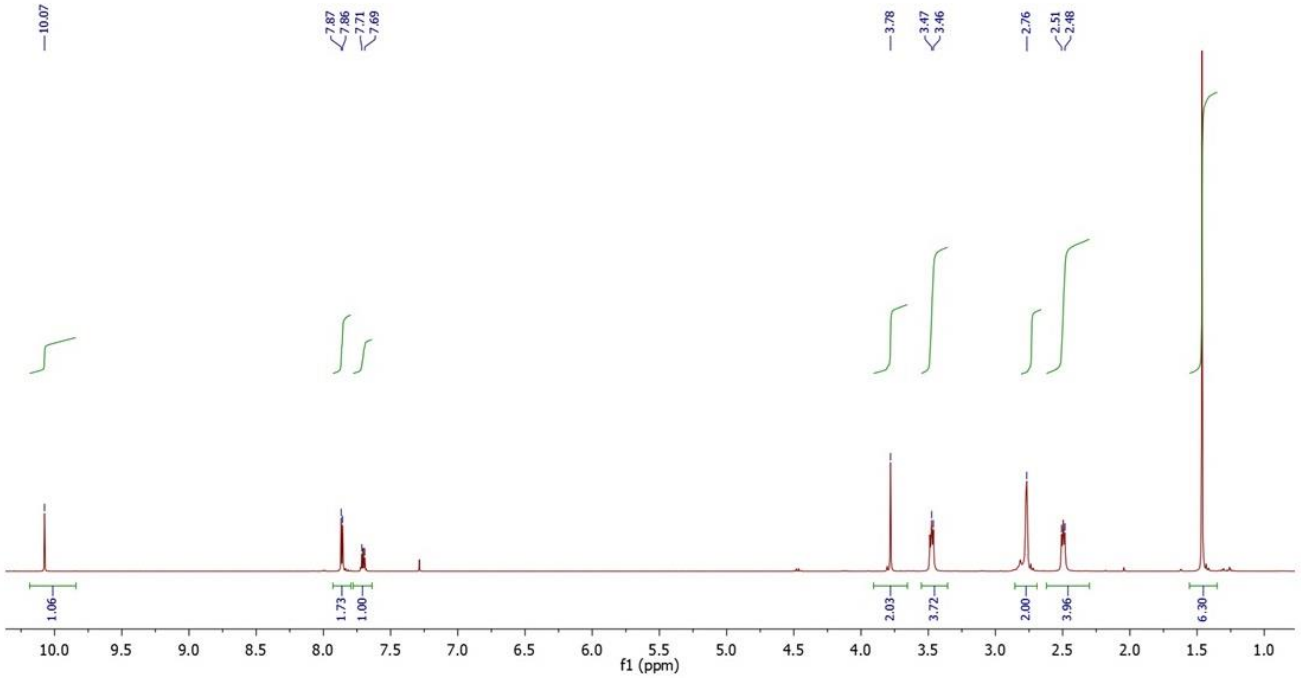

Figure S6. ^{1}H NMR spectrum of isocyanide **1a**.

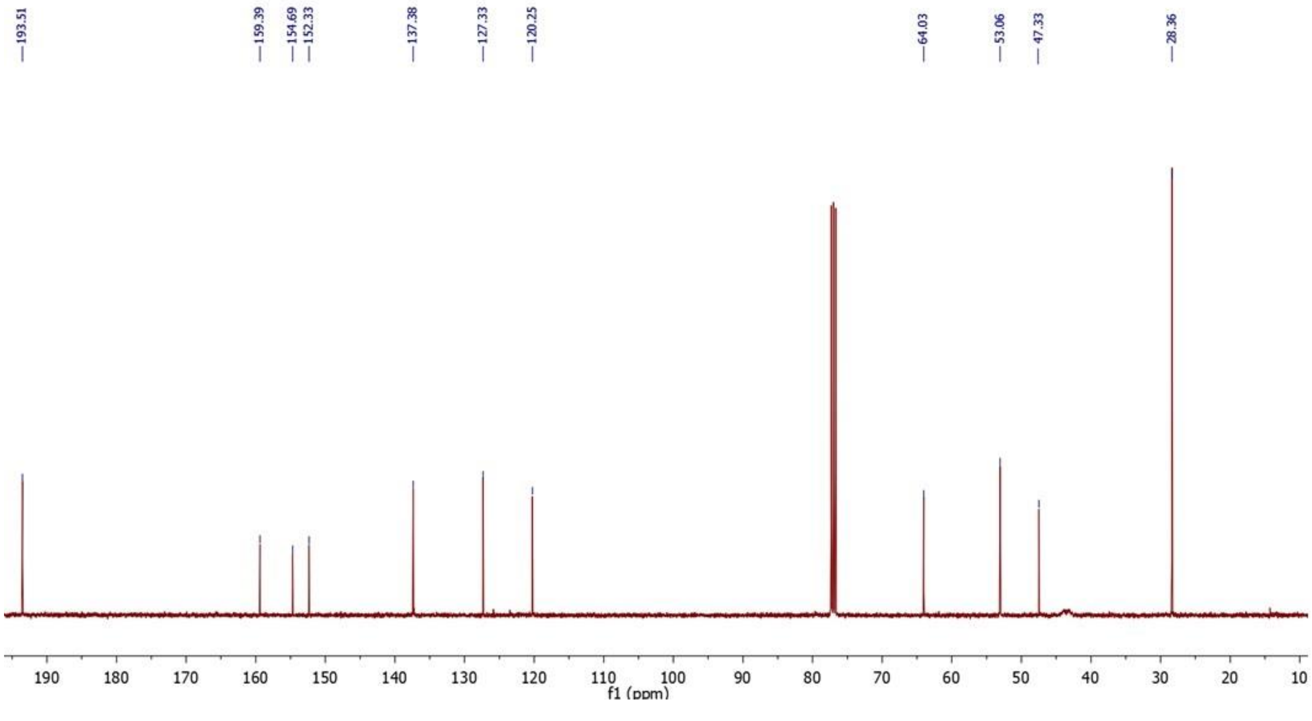

Figure S7. ^{13} NMR spectrum of isocyanide **1a**.

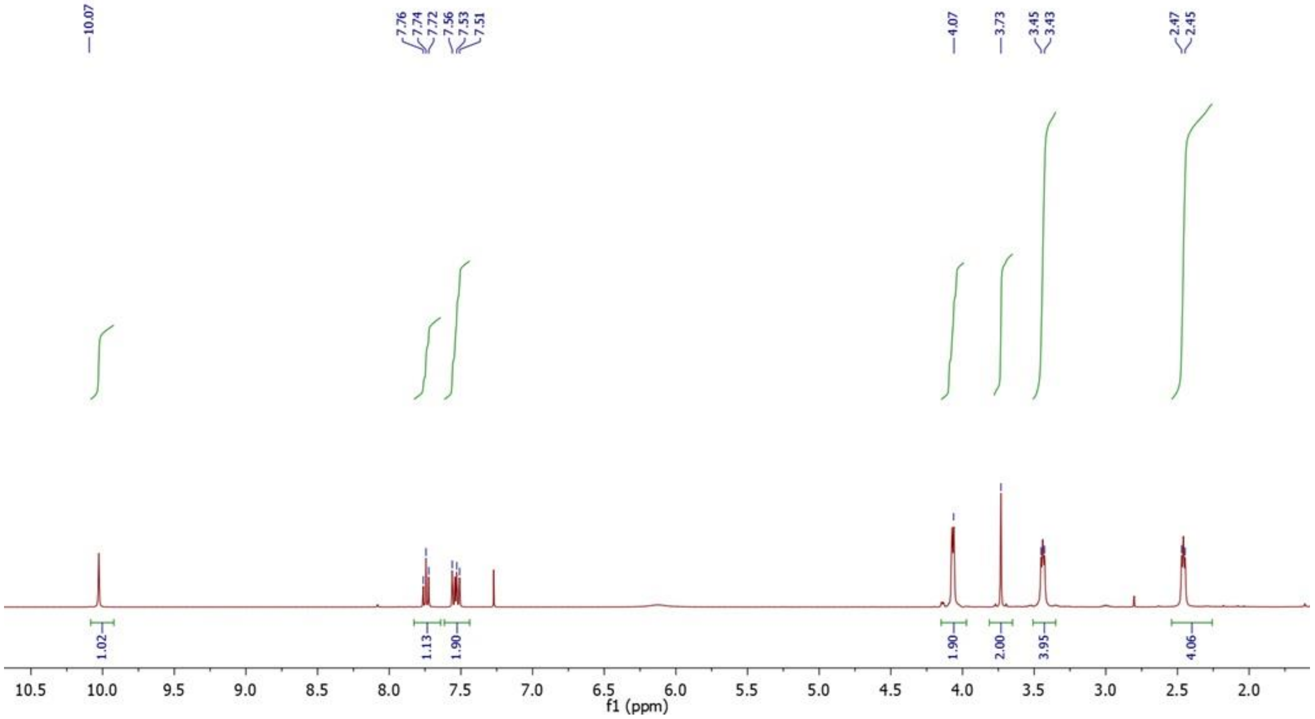

Figure S8. ^{1}H NMR spectrum of isocyanide **1b**.

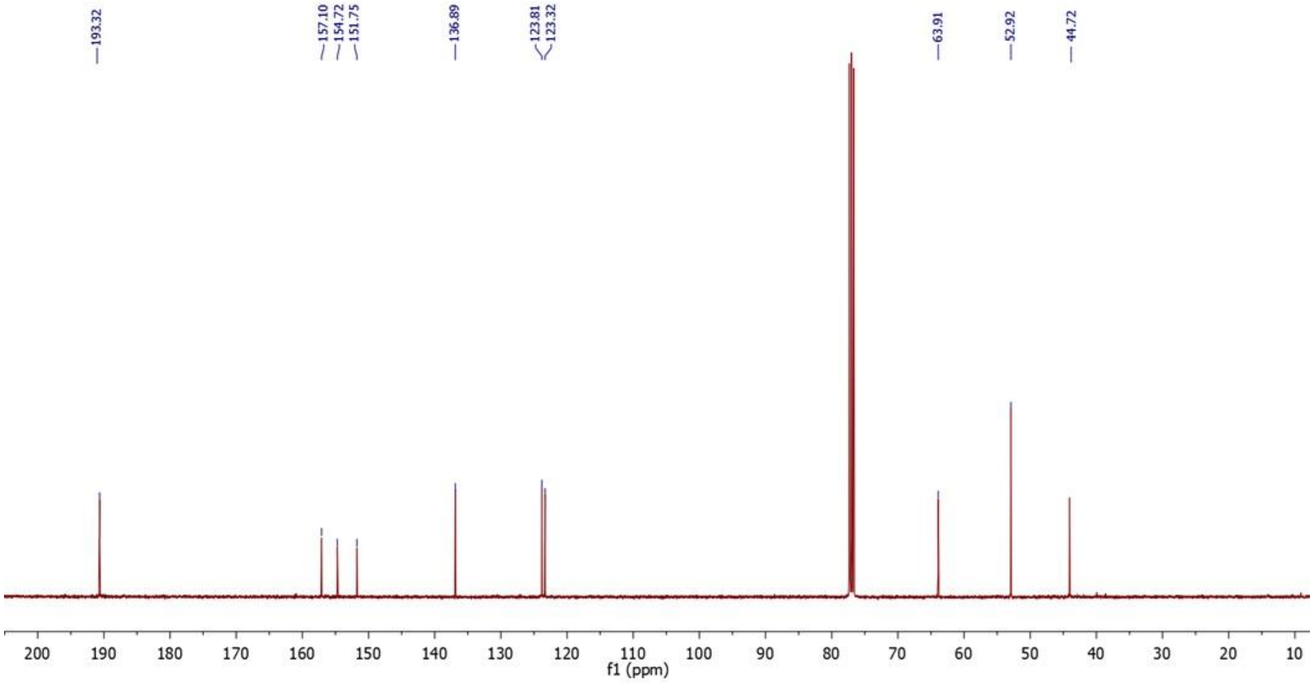

Figure S9. ^{13}C NMR spectrum of isocyanide **1b**.

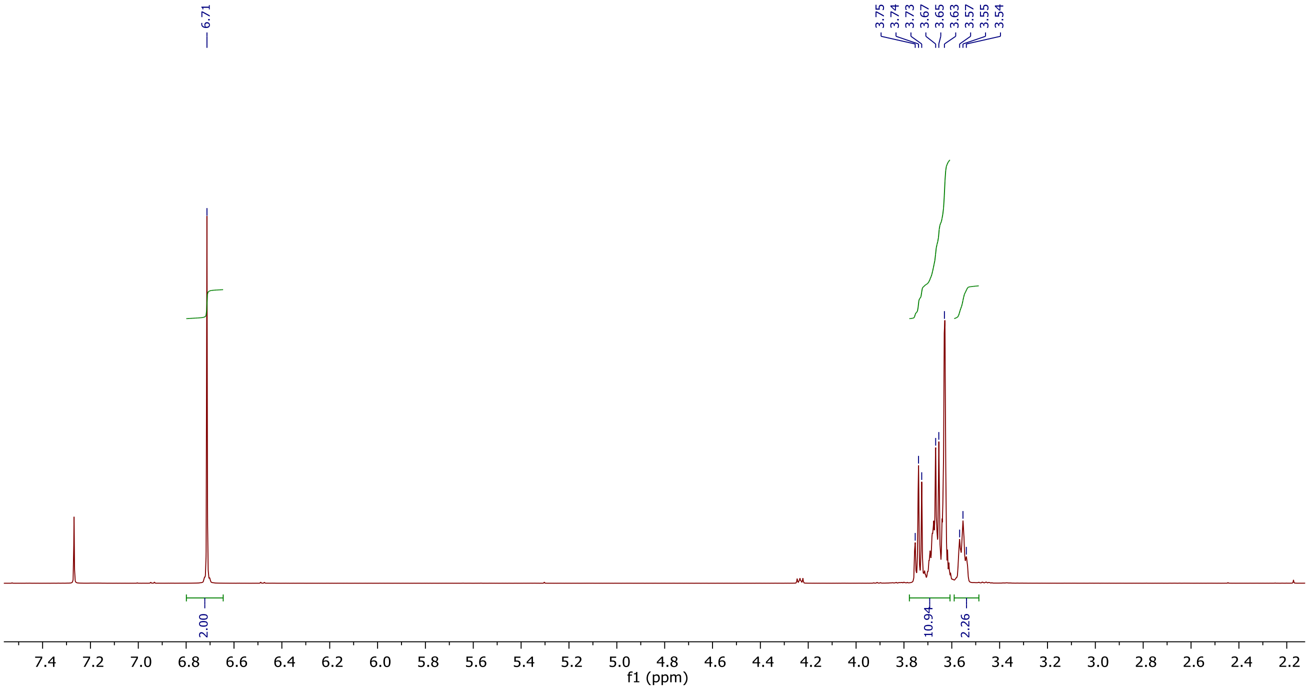

Figure S10. ^{1}H NMR spectrum of isocyanide **4** in CDCl3.

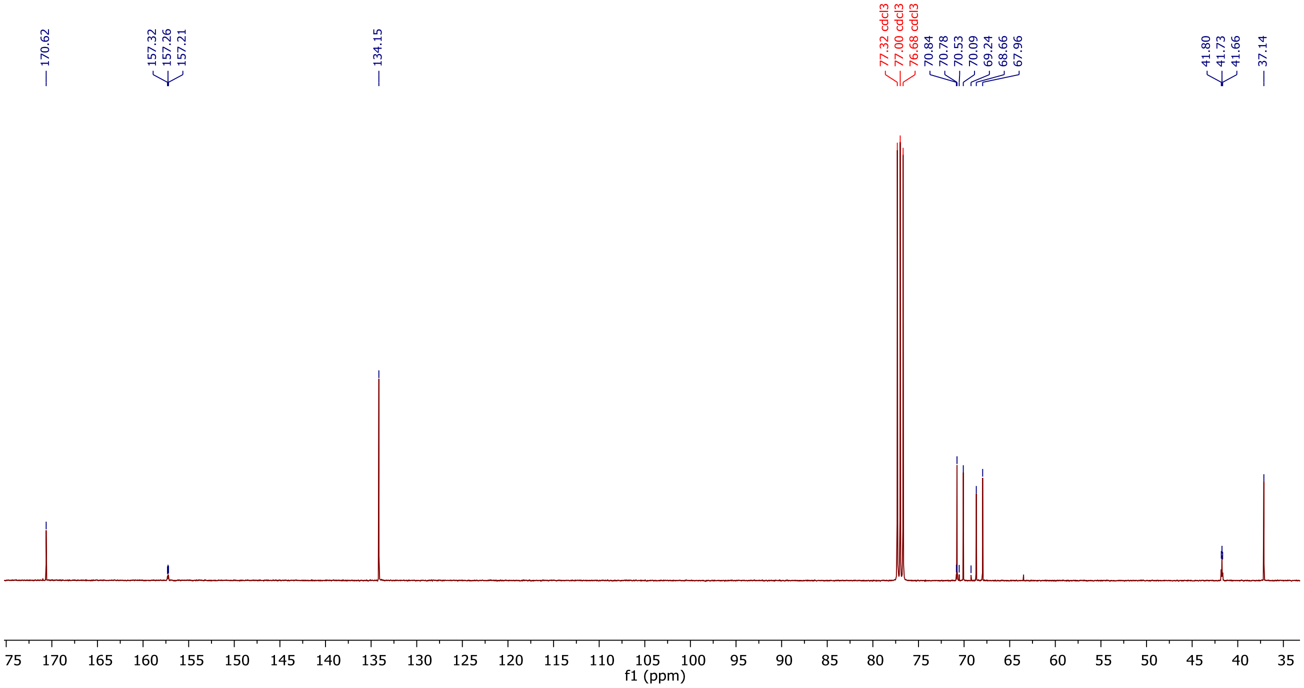

Figure S11. ^{13}C NMR spectrum of isocyanide **4** in CDCl3.

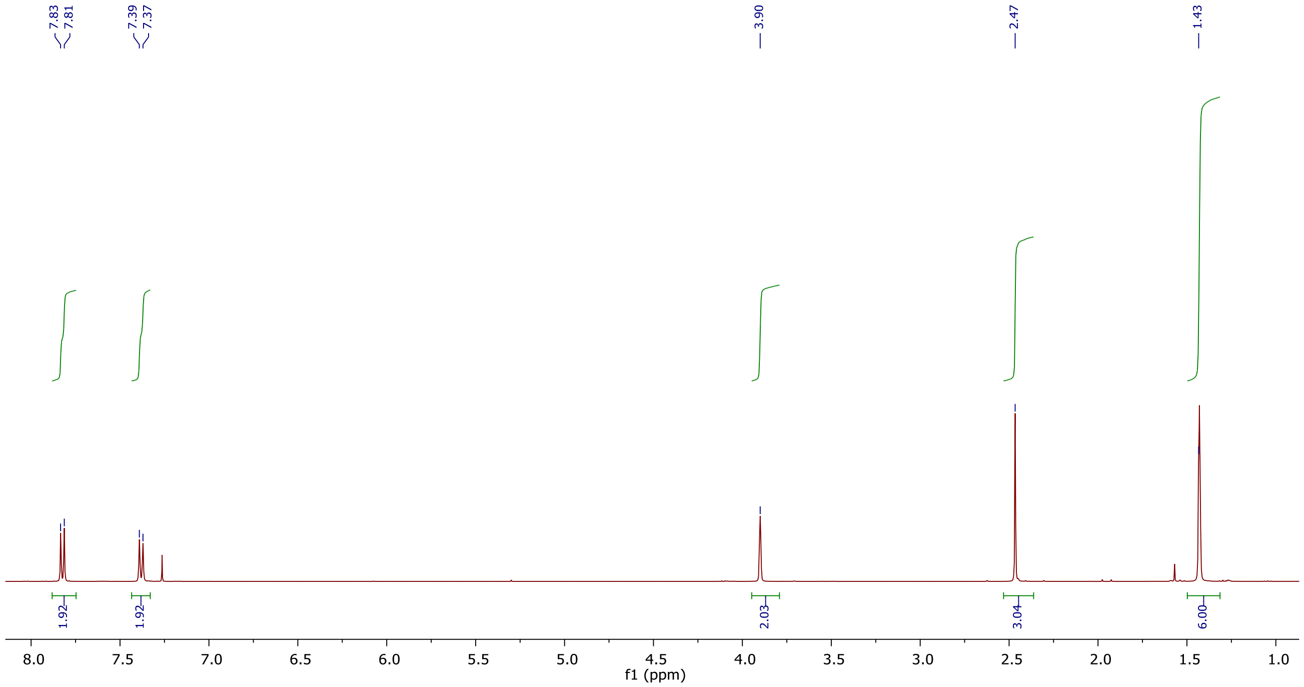

Figure S12. ^{1}H NMR spectrum of isocyanide **11a** in CDCl3.

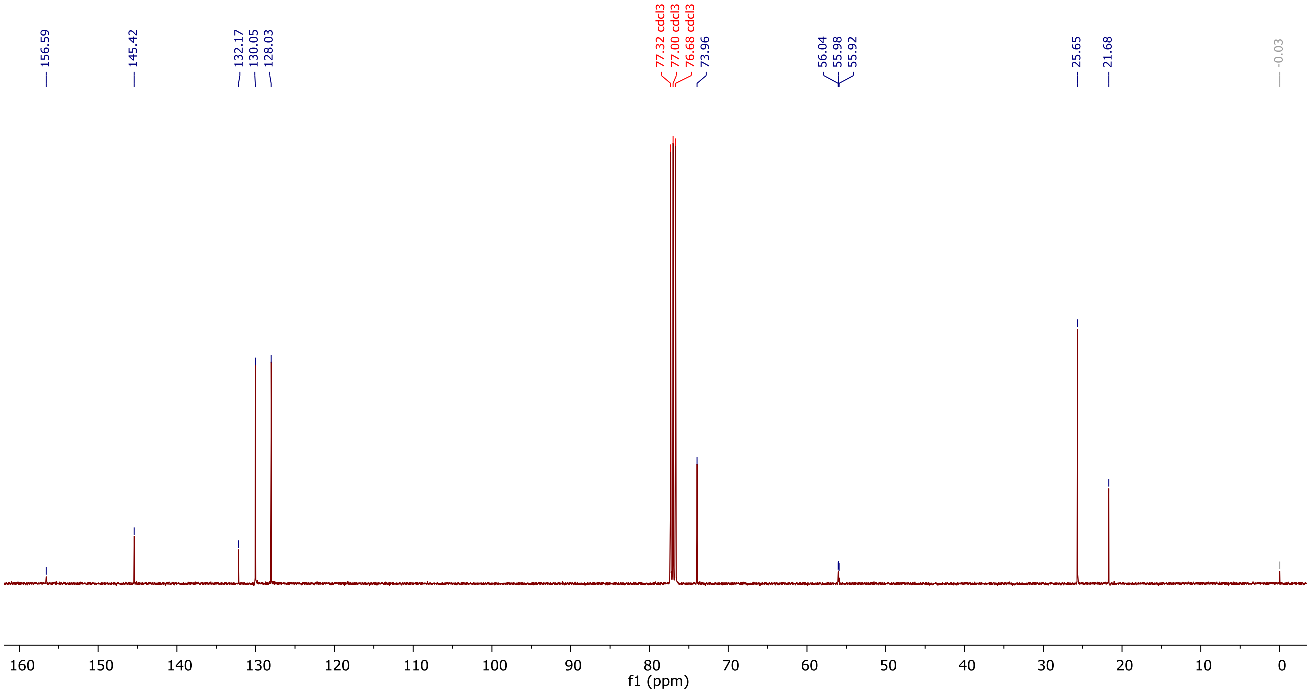

Figure S13. ^{13}C NMR spectrum of isocyanide **11a** in CDCl3.

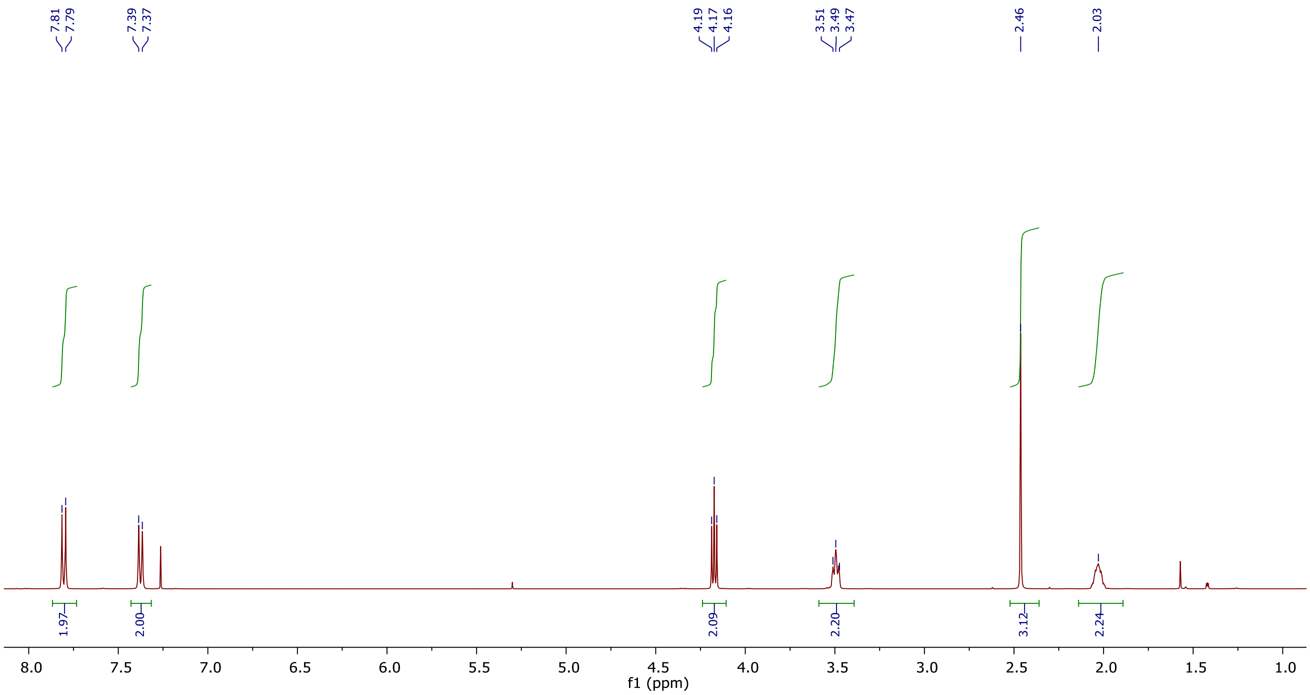

Figure S14. ^{1}H NMR spectrum of isocyanide **11b** in CDCl3.

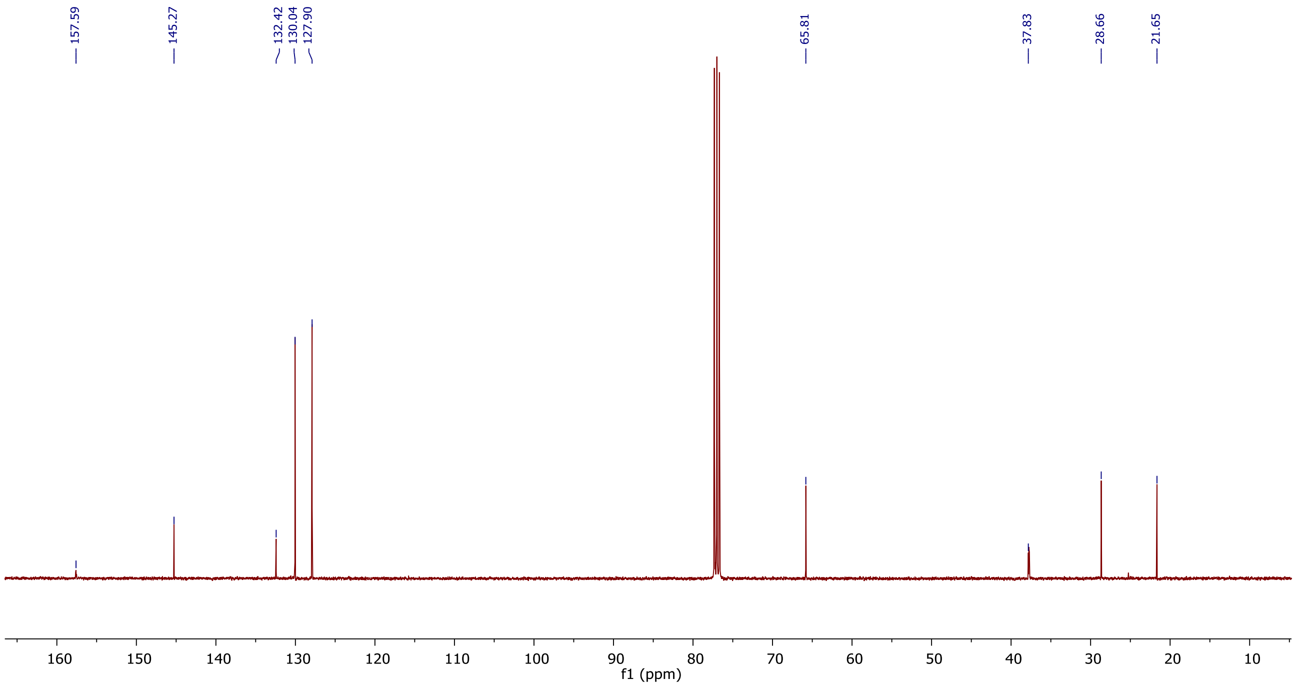

Figure S15. ^{13}C NMR spectrum of isocyanide **11b** in CDCl3.

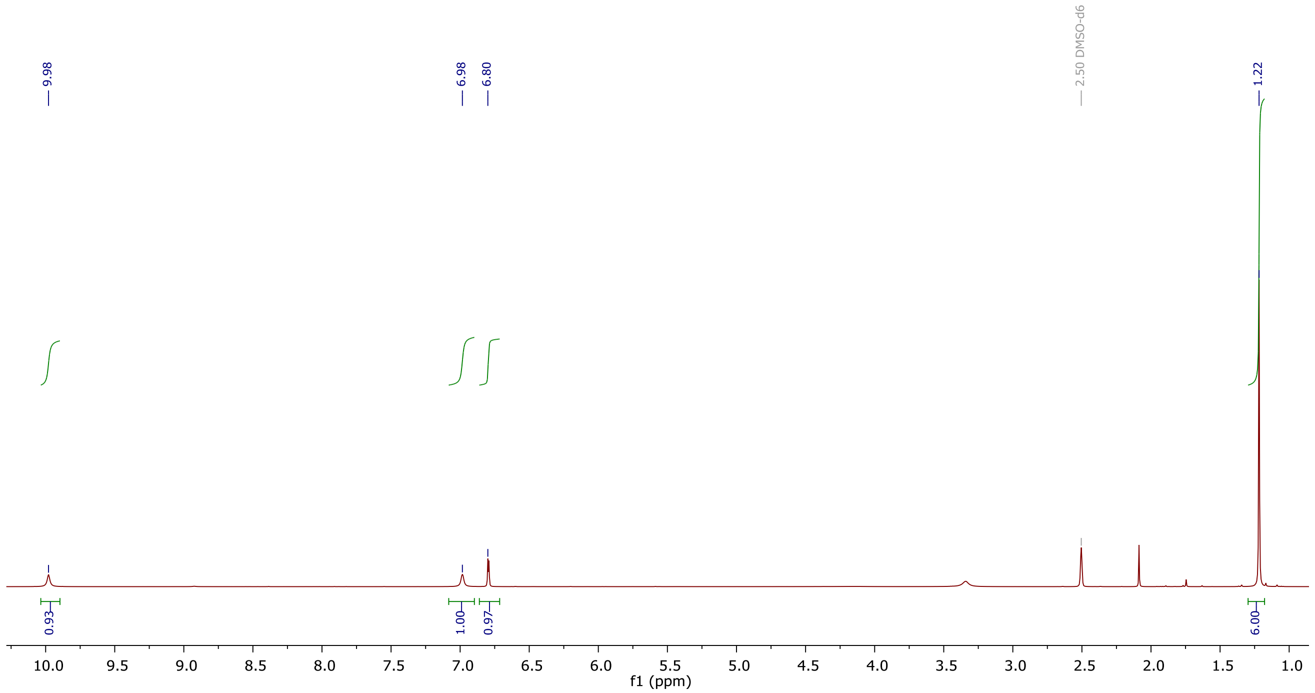

Figure S16. ^{1}H NMR spectrum of isocyanide **13** in DMSO-d_6.

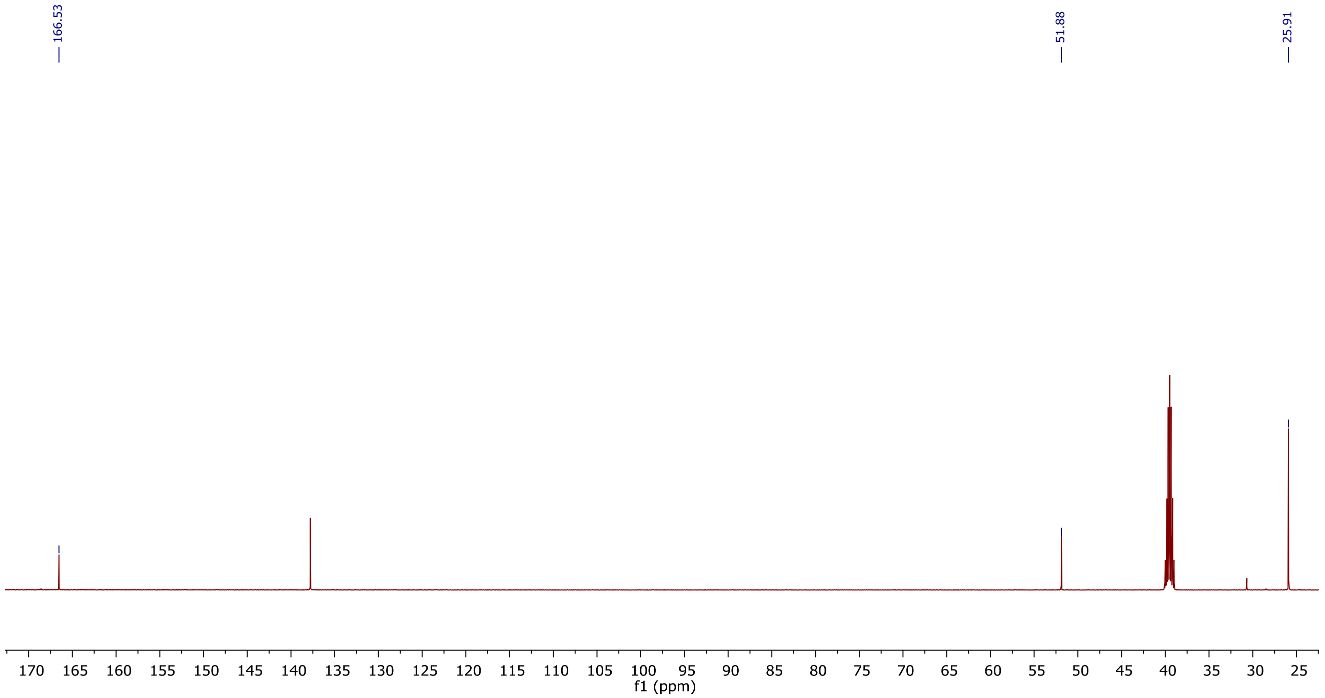

Figure S17. ^{13}C NMR spectrum of isocyanide **13** in DMSO-d_6.

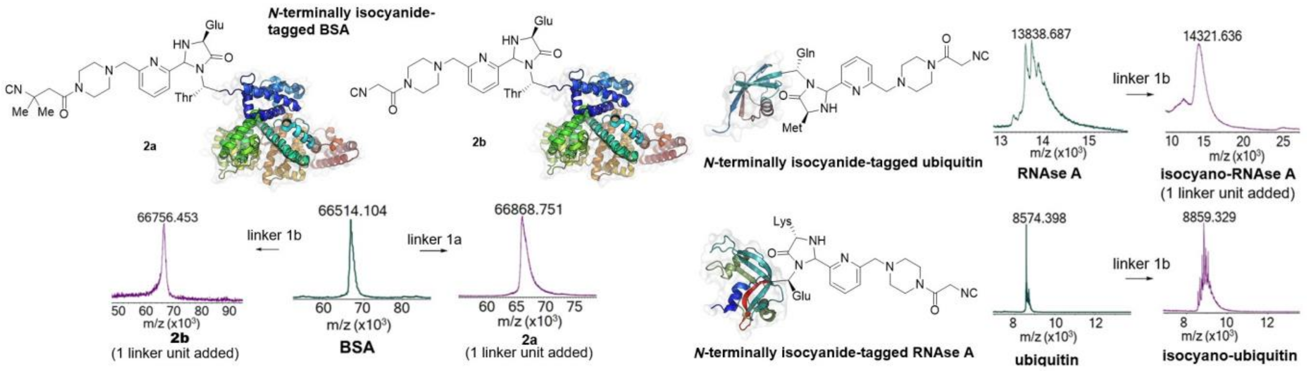

Figure S18. MALDI-TOF spectra of the N-terminally isocyanide-tagged proteins BSA, RNAse A and ubiquitin in comparison with the native proteins used as starting materials.

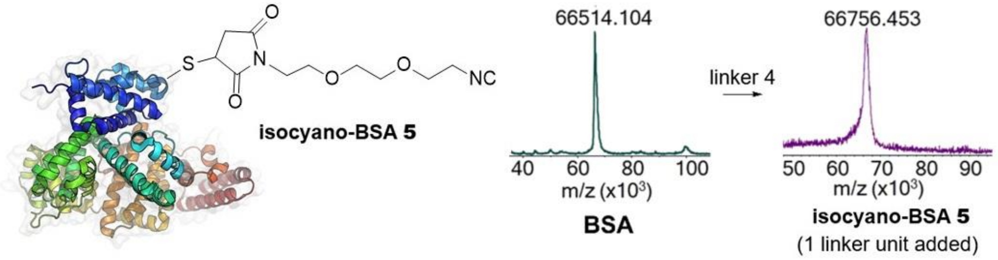

Figure S19. MALDI-TOF spectra of the site-selectively isocyanide-tagged BSA **5**.

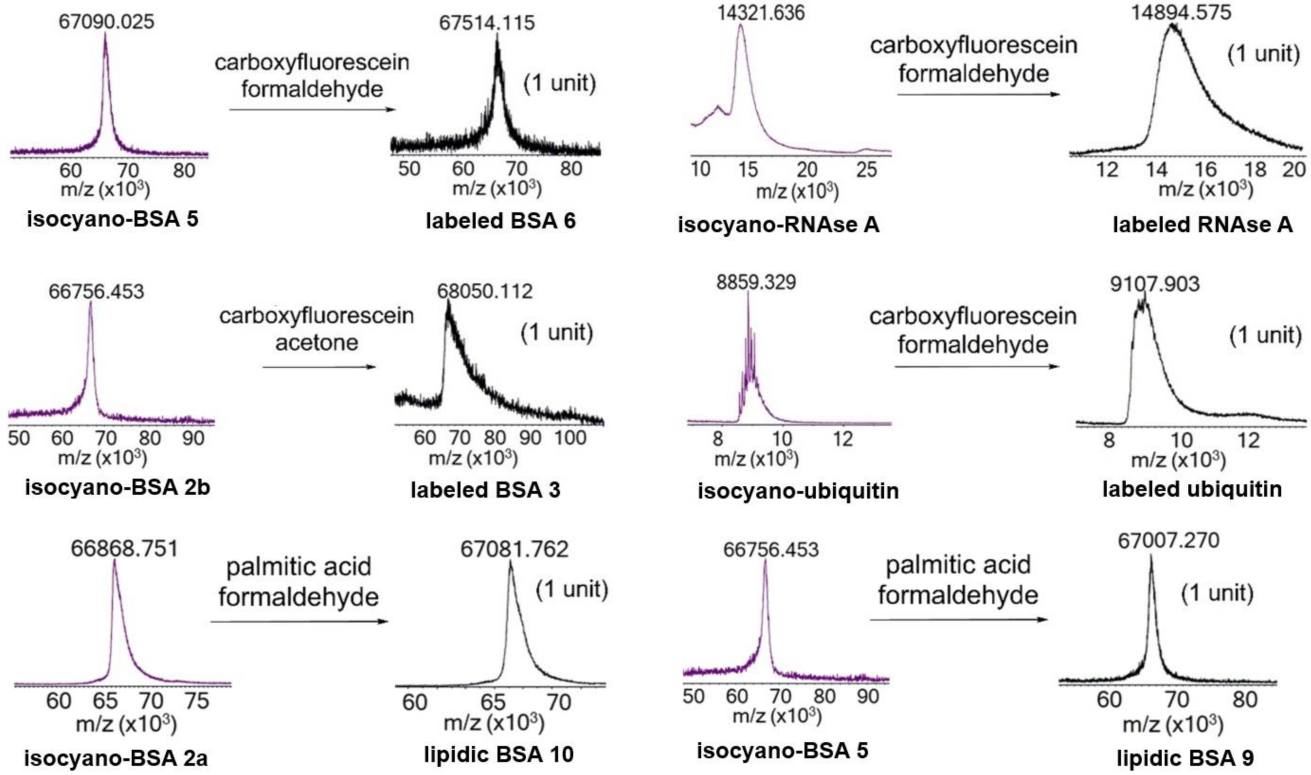

Figure S20. MALDI-TOF spectra of the fluorescently labeled and lipidic proteins in comparison with the isocyanoproteins used as starting materials in the Passerini bioconjugation.

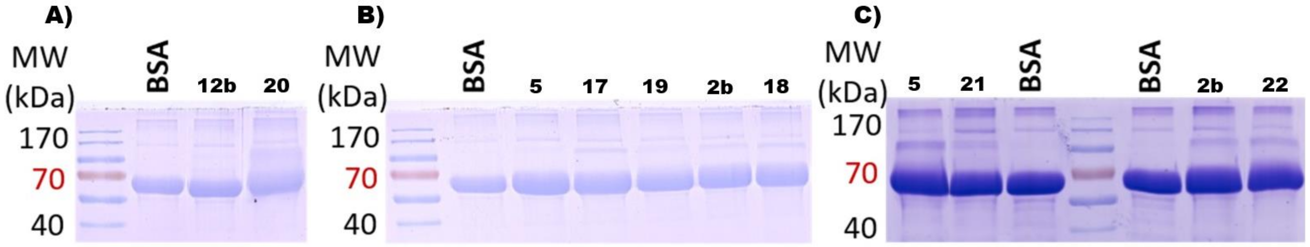

Figure S21. SDS-PAGE analysis of multivalent conjugates produced by the Passerini bioconjugation. A) Reaction of isocyano-BSA **12b** with sialic acid and PEG-aldehyde to produce the mutlimeric conjugate **20**. B) Reaction of isocyano-BSA **5** with sialic acid and lauryl aldehyde and PEG-aldehyde to produce the conjugates **17** and **19**, respectively. C) Reaction of isocyano-BSA **5** and **2b** with glycolipid GM3 and PEG-aldehyde to produce the conjugates **21** and **22**, respectively.

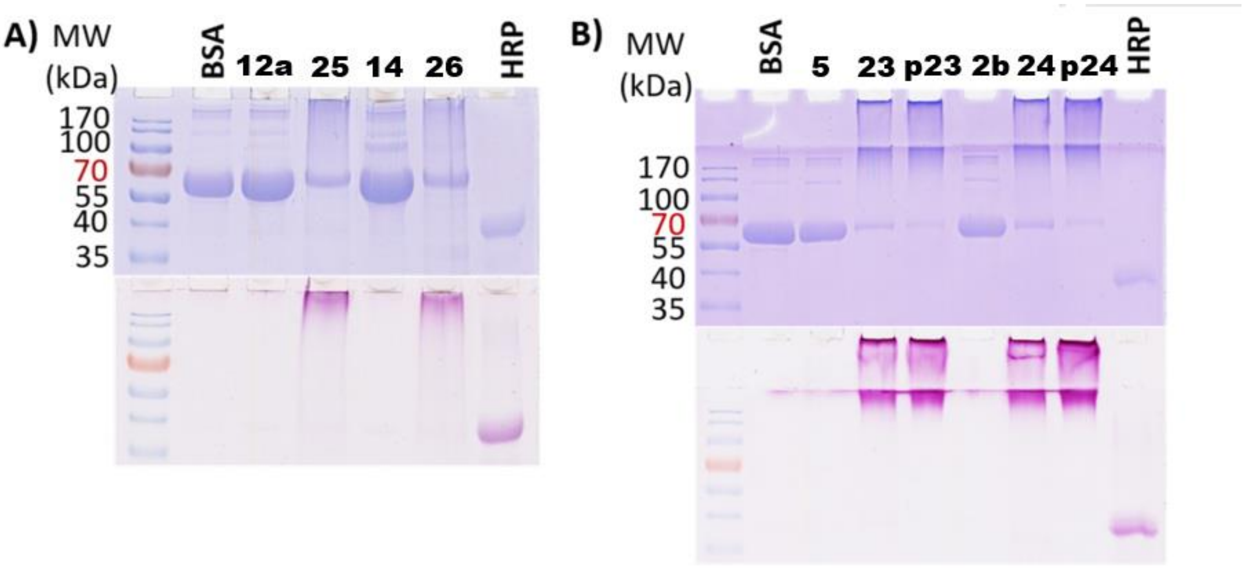

Figure S22. SDS-PAGE with Coomassie (above) and Fuchsin (below) staining of A) multimeric glycoconjugates **25** and **26** and B) site-selective glycoconjugates **23** and **24** before and after (**p23** and **p24**) SE-FPLC purification, in comparison with native BSA and the precursor isocyanoproteins. The glycoprotein HRP was employed as positive control for both staining.

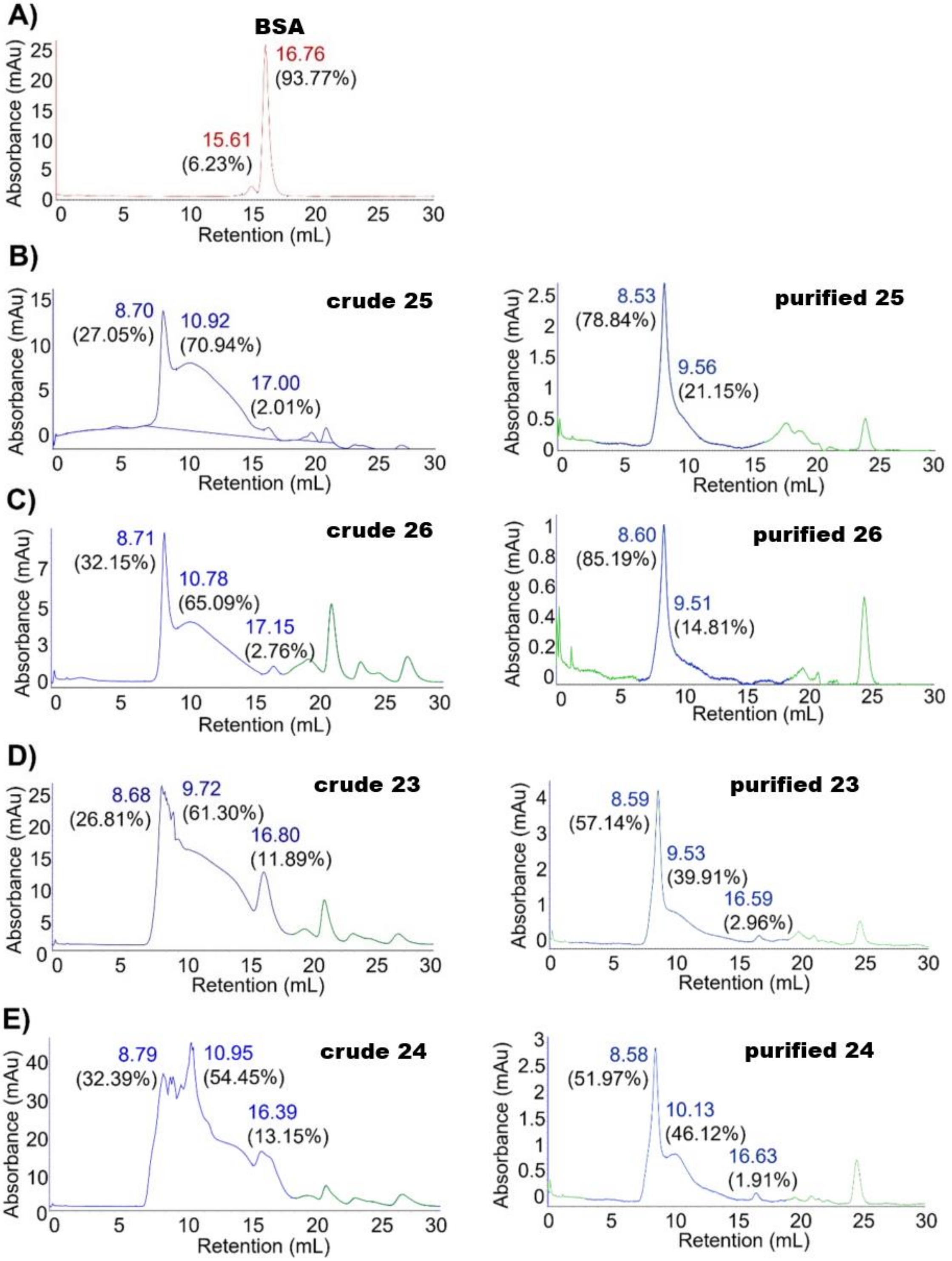

Figure S23. SE-FPLC traces (Superose® 6 column) of A) the non-conjugated BSA and the BSA-Pn14-MenC glycoconjugates B) **25**, C) **26**, D) **23** and E) **24** before and after purification. The samples were analyzed at 0.5 mL.min^{-1} of flow in PBS pH 7.4, 5 °C, with a UV detector. Crude glycoconjugate refers to the product treated only by diafiltration, while purified glycoconjugate refers to the product after a further purification by SE-FPLC.

Table S1. Average MW in ESI-TOF MS of isocyano-BSA **12a** and **14**.

BSA derivative	Expected mass addition (Da)	Average MW Theor. (Da)	Average MW Exp. (Da)	Error (%)[a]	Assignment
**12a**	+81.07	66512.7	66569.3	0.09	1 modification
	+81.07	66650.4	66653.8	0.005	2 modification
	+81.07	66734.9	66737.2	0.003	3 modification
	+81.07	66818.3	66835.9	0.03	4 modification
	+81.07	66917.0	66921.4	0.007	5 modification
**14**	+109.07	66540.7	66882.3	0.5	1 modification
	+109.07	66991.4	66991.7	0.0005	2 modification
	+109.07	67100.8	67100.7	0.0001	3 modification
	+109.07	67209.8	67204.4	0.008	4 modification
	+109.07	67313.5	67319.0	0.008	5 modification
	+109.07	67428.1	67428.1	0.00004	6 modification
	+109.07	67537.2	67537.9	0.001	7 modification
	+109.07	67647.0	67641.8	0.008	8 modification

[a]Relative error (%) = $|(\text{MW Theor.} - \text{MW Exp.})\ /\ \text{MW Theor.}| * 100$

Table S2. Structure of the repeating units of pneumococcal and meningococcal CPs.

**Polysaccharide**	**Repeating unit**
Streptococcus pneumoniae serotype 14 CPs	[β-D-Gal*p*-(1→4)]→6-β-D-Glc*p*NAc-(1→3)-β-D-Gal*p*-(1→4)-β-D-Glc*p*-(1→
Neisseria meningitidis serogroup C CPs	→9)-α-D-Neu*p*5Ac-[OAc-(→7/8)]-(2→

Table S3. Free protein percentage (UPP) and carbohydrate-protein ratio in the BSA-Pn14-MenC glycoconjugates

Glycoconjugate[a]	FPP%[b]	Oxo-CPs[c]/carboxy-CPs[d]/Protein[e]	CPs[b,c]/Protein[e]
**25**	2.0	1:1.3:2.5	1:1
purified **25**	0	ND	ND
**26**	2.7	1.8:1:5	1:2.8
purified **26**	0	ND	ND
**23**	11.9	3.5:1:2.3	2:1
purified **23**	2.9	ND	ND
**24**	13.1	2.3:1:1.5	2.2:1
purified **24**	1.9	ND	ND

[a]Purified glycoconjugate refers to SE-FPLC purification following the standard diafiltration process.
[b]Estimated by SE-FPLC in a Superose® 6 column, with manual integration of the area below the curve at the protein retention time
[c]CPs Pn14 quantification using the orcinol-sulfuric acid method
[d]CPs MenC quantification using the resorcinol method
[e]Protein quantification using Bradford method

References

[1] MacDonald, J. I.; Munch, H. K.; Moore, T.; Francis, M. B. One-step site-specific modification of native proteins with 2-pyridinecarboxyaldehydes. *Nat. Chem. Biol.* **11**, 326–331 (2015).
